# Does a Family Meetings Intervention Prevent Depression and Anxiety in Family Caregivers of Dementia Patients? A Randomized Trial

**DOI:** 10.1371/journal.pone.0030936

**Published:** 2012-01-27

**Authors:** Karlijn J. Joling, Harm W. J. van Marwijk, Filip Smit, Henriëtte E. van der Horst, Philip Scheltens, Peter M. van de Ven, Mary S. Mittelman, Hein P. J. van Hout

**Affiliations:** 1 Department of General Practice and the EMGO Institute for Health and Care Research, VU University Medical Center, Amsterdam, The Netherlands; 2 Department of Epidemiology and Biostatistics, EMGO Institute for Health and Health Care Research, VU University Medical Center, Amsterdam, The Netherlands; 3 Trimbos Institute (Netherlands Institute for Mental Health and Addiction), Utrecht, The Netherlands; 4 Department of Neurology, Alzheimer Center, VU University Medical Center, Amsterdam, The Netherlands; 5 Department of Psychiatry, New York University School of Medicine, New York, New York, United States of America; Federal University of Rio de Janeiro, Brazil

## Abstract

**Background:**

Family caregivers of dementia patients are at increased risk of developing depression or anxiety. A multi-component program designed to mobilize support of family networks demonstrated effectiveness in decreasing depressive symptoms in caregivers. However, the impact of an intervention consisting solely of family meetings on depression and anxiety has not yet been evaluated. This study examines the preventive effects of family meetings for primary caregivers of community-dwelling dementia patients.

**Methods:**

A randomized multicenter trial was conducted among 192 primary caregivers of community dwelling dementia patients. Caregivers did not meet the diagnostic criteria for depressive or anxiety disorder at baseline. Participants were randomized to the family meetings intervention (n = 96) or usual care (n = 96) condition. The intervention consisted of two individual sessions and four family meetings which occurred once every 2 to 3 months for a year. Outcome measures after 12 months were the incidence of a clinical depressive or anxiety disorder and change in depressive and anxiety symptoms (primary outcomes), caregiver burden and quality of life (secondary outcomes). Intention-to-treat as well as per protocol analyses were performed.

**Results:**

A substantial number of caregivers (72/192) developed a depressive or anxiety disorder within 12 months. The intervention was not superior to usual care either in reducing the risk of disorder onset (adjusted IRR 0.98; 95% CI 0.69 to 1.38) or in reducing depressive (randomization-by-time interaction coefficient = −1.40; 95% CI −3.91 to 1.10) or anxiety symptoms (randomization-by-time interaction coefficient = −0.55; 95% CI −1.59 to 0.49). The intervention did not reduce caregiver burden or their health related quality of life.

**Conclusion:**

This study did not demonstrate preventive effects of family meetings on the mental health of family caregivers. Further research should determine whether this intervention might be more beneficial if provided in a more concentrated dose, when applied for therapeutic purposes or targeted towards subgroups of caregivers.

**Trial Registration:**

Controlled-Trials.com ISRCTN90163486

## Introduction

Caring for a family member with dementia can be a stressful experience and has been associated with negative outcomes such as depression and anxiety. According to one study, almost half of the caregivers developed depression diagnosed according to research diagnostic criteria within one year after the initial assessment [1], [2]. Previously, we estimated that spouses of patients with dementia have a fourfold higher risk of depression than spouses of non-demented persons [3]. The incidence of anxiety among dementia caregivers has received less attention, but a recent review showed that clinically significant anxiety affects about a quarter of caregivers of people with dementia and was more common than in matched controls [4].

Given the large number of caregivers who may suffer from clinical depression or anxiety, providing adequate treatment for all of them would place a heavy burden on health care resources and might not be feasible. Alternative strategies, such as prevention, can potentially be a more cost effective approach. Preventive interventions have proven to be effective in reducing the incidence of anxiety and depressive disorders in non-caregiving populations [5]–[8] and are most likely to be effective when targeted at those with a high a priori risk of developing the disorder [9], [10]. This can be achieved by focusing on people exposed to established risk factors for a disorder (selective prevention), as is the case in the population of dementia caregivers. Thus, the literature suggests that a preventive intervention might be an effective strategy for this target group.

Over the last decades, various strategies for supporting caregivers have been developed. Systematic reviews and meta-analyses indicate mixed results with respect to the effectiveness of these interventions on caregiver mood or burden [11], [12]. Information and support alone may be helpful, but appears to address the psychological needs of caregivers only marginally [13], [14]. Programs that demonstrate beneficial effects involve both patients and their families, are more intensive and are designed to meet each caregiver's individual needs [13], [15]. Results of the NYU Caregiver Intervention (NYUCI), a multi-component intervention that included family counseling, individual counseling sessions, support group participation and continuous availability of ad hoc counseling have demonstrated that counseling and support interventions designed to mobilize support of naturally existing family networks, appear to be effective in reducing depressive symptoms in caregivers [16], [17] and in delaying nursing home placement of the dementia patient [18], [19]. However, it is unclear whether family meetings alone have preventive effects. Therefore, in this study we investigated whether structured family meetings are more effective than usual care in the prevention of depression or anxiety disorders in caregivers. We also evaluated the effects on the severity of depressive and anxiety symptoms, caregiver burden and quality of life of the caregiver.

## Methods

The protocol for this trial and supporting CONSORT checklist are available as supporting information; see Checklist S1 and Protocol S1.

### Participants

Caregivers and patients were recruited through memory clinics (n = 91), services delivering case management (n = 79), general practices, home care settings and meeting centers for people with dementia and their caregivers (n = 22) in the Netherlands. Caregivers were eligible if they were the primary family caregiver of a community dwelling relative with a clinical diagnosis of dementia and had at least one other family member or friend available to participate in the family meetings. If there was more than one family caregiver caring for the patient, the primary caregiver was identified as the person who coordinated the caring process, usually the person who spends most hours on caregiving tasks. Caregivers were excluded when 1) they met the criteria for a clinical depressive or anxiety disorder as measured with the Mini International Neuropsychiatric Interview (MINI) [20], 2) the patient was scheduled to move into a nursing home, 3) they presented with severe somatic or psychiatric co-morbidity which would significantly impair cooperation with the study, and 4) they had insufficient proficiency in the Dutch language thus hampering adequate participation in meetings and interviews.

### Ethics

The Medical Ethics Committee of the VU University Medical Center approved the study protocol. Written informed consent was obtained from all participants.

### Intervention

Caregivers randomized to the intervention group were invited to participate in six in-person counseling sessions: one individual preparation session, followed by four structured meetings that included their relatives and/or friends (family meetings), and one additional individual evaluation session. The family meetings were held once every 2 to 3 months in the year following enrollment in the program based on pilot experience with some families prior to the study.

#### Preparation meeting

The first individual session was aimed to prepare the caregiver for the family meetings and to propose the idea of seeking help from family and friends.

#### Family meetings

The aim was to offer psycho-education, teach problem solving techniques and mobilize the existing family networks of the patient and primary caregiver in order to improve emotional and instrumental support. The content of the sessions was guided by the needs of the caregiver. During the first family meeting the purpose of the meetings, the protocol, ground rules and the counselor's role were explained to the caregiver and the family. Relevant issues were identified (e.g. management of patient behavior problems, coping with feelings of guilt) and the counselor motivated the family to form ideas to help the caregiver and to delegate tasks. The follow up meetings reviewed the previous session, previous commitments and the progress of tasks. Ad hoc telephone counseling from the same counselor was available to caregivers and their families beyond the scheduled sessions.

#### Evaluation session

After the final family session, an individual session was held to evaluate the caregiver's satisfaction with the intervention program and to start additional support when requested.

The counselors who led the family meetings had an advanced degree in nursing, social work, psychology or an allied profession and were trained prior to the study by the research team. One counselor was assigned to each caregiver to establish an ongoing relationship with a person familiar with the situation. The family meetings were audio taped for supervision and reviewed randomly and on request to give feedback to the counselors. To encourage and evaluate protocol adherence, after a family session, the counselor filled in a standardized form and was contacted by the researcher (KJ) individually to monitor and discuss difficulties. For more detail about the intervention, see Joling et al. 2008 [21].

#### Usual care

Caregivers randomized to the control condition received care as usual. Usual care in the Netherlands may consist of a range of health care and welfare services and can differ across participants. However, family meetings are rarely organized and never in a structured way or with follow-up sessions. They also tend to focus on providing clinical information and not on increasing family support and relieving the caregiver. Usual care participants were free to use all types of care, including community-based mental health services or support resources other than family meetings at any time throughout the 12 months follow-up, therefore reflecting standard care. The participants' use of health care services and their participation in family meetings was recorded.

### Objective

To determine whether a family meetings intervention prevents the development of anxiety and depressive disorders superior to usual care. The design of this study has been described in detail elsewhere [21].

### Primary & secondary outcomes

#### Primary outcomes

Major depressive disorder and anxiety disorders were assessed every 3 months after enrollment with the Mini International Neuropsychiatric Interview (MINI) [20]. The MINI is a short, structured diagnostic interview for DSM-IV mental disorders and can be used for psychiatric evaluation and outcome tracking. The severity of symptoms of depression was measured every 6 months with the Center for Epidemiologic Studies Depression Scale (CES-D) and with the Hospital Anxiety and Depression Scale- Anxiety subscale (HADS-A) for anxiety. The CES-D consists of 20 items, with a total score ranging between 0 and 60. Higher scores indicate greater psychological distress, and scores of 16 and above indicate the presence of clinically significant depression [22]. The HADS-A consists of 7 items, with total scores ranging between 0 and 21. A score of 8 or higher suggests the presence of clinically significant anxiety [23], [24].

#### Secondary outcomes

Secondary outcomes were assessed every 6 months and included: five dimensions of caregiver burden (disrupted time, financial problems, lack of family support, health problems, and caregiver's self-esteem) measured with the Caregiver Reaction Assessment (CRA) [25], and health-related quality of life using the Short Form-12 item questionnaire (SF-12) [26].

### Sample size

The sample size calculation was based on the expected effects of the intervention on the main outcome measures, incidence of a depression or anxiety disorder. The yearly incidence of disorders among caregivers at risk was estimated at 30% [2]. The trial was powered to detect a 20% decrease in the incidence. We calculated that 73 participants per group would be needed, assuming a 2-sided test, an alpha of 0.05 and a power of 80%. With a drop out of 20%, at least 182 participants would be needed.

### Randomization & blinding

After informed consent and baseline measurement, dyads of patients and their primary family caregiver were randomized by an independent researcher, stratified by recruitment center, in blocks of four to either usual care or the family meetings intervention. The interviewers who measured the outcomes were blinded to randomisation status. The participants and the counselors conducting the family meetings were aware of the intervention assigned.

### Statistical analyses

We investigated if randomization had resulted in a balanced distribution of prognostically important variables across the conditions. In addition, we compared the baseline characteristics of dropouts and those who completed the 12-month measurement by performing logistic regression analysis. Data were primarily analyzed on the basis of the intention-to-treat principle (ITT), which means that all participants were analyzed in the group to which they were randomized. This analysis requires imputation of missing observations. The missing values on the outcomes measures were imputed using multiple imputation by chained equations. In contrast to other imputation techniques, this method minimally alters variance of data, thus providing the best estimates of missing data, at least until 50% of the data are missing [27]. To estimate to what extent the intervention was more successful in reducing the risk of depressive and anxiety disorders than usual care, we first performed an unadjusted Poisson regression analysis of the clinical depression or anxiety cumulative incidence (1 = became disordered and 0 = remained disorder free over the last year) on the treatment indicator (0 = usual care, 1 = intervention). In this manner we obtained a crude incidence rate ratio (IRR) which describes the difference in incidence rate in the intervention relative to usual care. The superiority of the intervention would be supported if the IRR falls below 1 and would be significant at P<0.05, 2-tailed. Estimates of the intervention effects on the continuous outcome measures were obtained from linear mixed models. These take into account the repeated measurements for each subject. The randomization status, time of measurement and randomization-by-time interaction were included as fixed effects in the models. Multilevel modeling was used to analyze the repeated measurements. The models included a random intercept for each site and a random intercept and slope for each patient. We assessed the overall effect of the intervention by testing the interaction between randomization and time of measurement. To adjust for selection-bias, variables with significant baseline differences and a significant association with outcome (HADS-A baseline score) were incorporated as covariates in all analyses. All analyses were performed while taking into account the hierarchical structure of data, with caregivers nested in recruitment sites. The models were fitted in Stata (version 11) using the xtmixed command. Because placement and death of the patient might affect the outcomes, the ITT analyses were replicated removing the caregivers where the patient either died or was institutionalized during the study. The results of the intention-to-treat analyses were compared with the results of the per protocol analysis. This type of analysis can assess whether protocol violations have caused bias. In the per-protocol analyses, outcomes of the participants who attended at least three family meetings were compared with the outcomes of the usual care group.

Finally, we performed ancillary analyses to investigate the possible modification of the treatment effect by caregiver gender, dementia severity, initial depressive or anxious symptoms within the caregivers, lack of family support, and recruitment via sites offering intensive support resources. These effect modification analyses were conducted to determine whether caregivers with certain baseline characteristics would benefit (more) from family meetings. The analyses were performed with the SPSS (version 15.0) and Stata (version 11) statistical packages.

## Results

### Participant flow and recruitment

**Figure 1:**
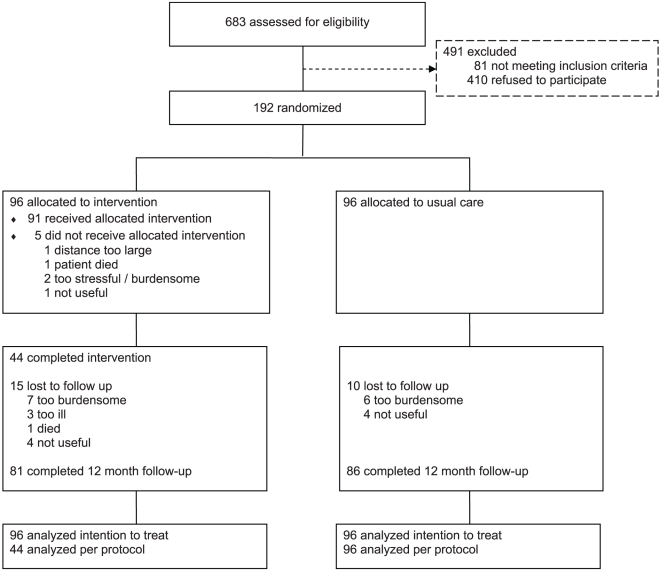
Flow diagram of the study sample.

Participants were recruited from November 2007 to November 2009. Of the caregivers assessed for eligibility, 192 met all inclusion criteria and were willing to participate (Figure 1). Reasons for exclusion were ‘patient not diagnosed with dementia’ (n = 10), ‘no other family member or friend available to participate in the family meetings’ (n = 16), ‘insufficient command of the Dutch language’ (n = 9), ‘patient was (scheduled to be) institutionalized’ (n = 39), ‘caregiver had a clinical depressive or anxiety disorder at intake’ (n = 7).

A substantial number of 410 caregivers refused to participate. Most refusals were due to claiming a lack of need for this intervention (n = 202). These caregivers already used other services, said they could (still) manage on their own or did not expect to benefit from the intervention. Other reasons for refusal included: too burdensome (n = 85), practical reasons (n = 33), resistance of the family or patient (n = 21), not willing to burden their family (n = 19), difficulties with the randomized design (n = 3) and reason unknown (n = 47). There were no significant differences between the dyads of caregivers and patients who declined participation and the participating dyads in gender, caregiver-patient relation and the type of service they were recruited from. Patients recruited from memory clinics were younger than those recruited from casemanagement (difference in mean = −4.58, 95% CI −7.68 to −1.48, p = 0.002) and primary care settings (difference in mean = −7.04, 95% CI −11.83 to −2.26, p = 0.002)

### Baseline characteristics

Table 1 presents the socio-demographic and clinical characteristics of the caregivers and patients at baseline. Caregivers and patients in the intervention group were significantly younger (difference in mean patients' age = 3.85 , 95% CI 1.372 to 6.328, t = 3.07 with 190 df, p = 0.002 and difference in mean caregivers' age = 3.37, 95% CI 0.447 to 6.302, t = 2.27 with 190 df, p = 0.024) and caregivers had higher HADS-A scores (difference in mean = −1.24, 95% CI −2.219 to −0.266, t = −2.51 with 188 df, p = 0.013) at baseline than participants in the usual care group. The HADS-A baseline score was also a significant predictor of outcome and was therefore incorporated as covariate in the analyses.

**Table 1:** Baseline demographic and clinical characteristics of the caregivers and patients.

	Caregiver		Patient	
	Intervention (n = 96)	Usual care (n = 96)	Intervention (n = 96)	Usual care (n = 96)
Age, M (SD)	67.8 (9.8)*	71.2 (10.7)	72.8 (9.1)*	76.7 (8.3)
Female gender, n (%)	67 (69.8)	68 (70.8)	30 (31.3)	32 (33.3)
Spouse of the patient, n (%)	92 (95.8)	89 (92.7)		
Living with patient, n (%)	93 (96.9)	91 (94.8)		
Educational level, n (%)				
Elementary/Lower	28 (29.2)	34 (35.4)	42 (43.8)	44 (45.8)
Secondary	37 (38.5)	30 (31.3)	30 (31.3)	28 (29.2)
Higher/University	29 (30.2)	32 (33.3)	24 (25.0)	22 (22.9)
Chronic diseases				
0 (%)	32 (33.3)	28 (29.2)	25 (26.0)	26 (27.1)
1 or more (%)	63 (65.6)	68 (70.8)	70 (72.9)	70 (72.9)
CES-D (0–60), M (SD)	12.1 (7.9)	10.8 (7.1)		
≥16, n (%)	31 (32.3)	23 (24.0)		
HADS-A (0–21), M (SD)	6.1 (3.4)*	4.8 (3.5)		
≥8, n (%)	32 (33.3)	23 (24.0)		
CRA Disrupted time (5–25) , M (SD)	15.6 (4.0)	15.2 (4.3)		
CRA Financial problems (3–15) , M (SD)	6.5 (1.8)	6.4(1.8)		
CRA Lack of family support (5–25) , M (SD)	12.8 (4.0)	12.2 (3.9)		
CRA Health problems (4–20) , M (SD)	10.3 (2.8)	9.9 (3.1)		
CRA Caregiver's self-esteem (7–35) , M (SD)	26.9 (3.5)	26.9 (3.7)		
Can caregiver leave the patient alone?				
No	13 (13.5)	8 (8.3)		
Yes, but not unlimited	59 (61.5)	65 (67.7)		
Yes, unlimited	23 (24.0)	23 (24.0)		
ADL independencies (out of 6), M (SD)			5.1 (1.4)	5.3 (1.1)
IADL independencies (out of 7), M (SD)			2.7 (1.8)	2.6 (1.5)
MMSE (0–30), M (SD)			21.4 (4.9)	21.7 (5.6)
NPI-Q (0–36), M (SD)			8.5 (5.4)	9.5 (6.3)
Type of dementia				
Alzheimer disease			54 (56.3)	56 (58.3)
Other			38 (39.6)	35 (36.5)
Type not specified/unknown			4 (4.2)	5 (5.2)

M: Mean; SD: Standard deviation; CES-D: Center for Epidemiologic Studies Depression Scale; HADS-A: Hospital Anxiety and Depression Scales; CRA: Caregiver reaction assessment scale; (I)ADL: (Instrumental) activities of daily living; MMSE: Mini Mental State Examination; NPI-Q: Neuropsychiatric Inventory- Questionnaire.

*significant difference with usual care group (p<0.05).

### Numbers analyzed

Follow-up data were collected for twelve months after enrollment from 81/96 (84%) intervention and 86/96 (90%) usual care group participants. Loss to follow-up was significantly associated with poorer perceived physical health of the caregiver at baseline (OR = 0.959, 95% CI 0.923 to 0.996). In accordance with the intention-to-treat principle, missing follow-up data were imputed and all participants were included in the analyses.

### Primary outcomes

#### Incidence of depression and anxiety

An incident depressive or anxiety disorder occurred in 38 participants in the intervention group and 34 participants in the usual care group (unadjusted IRR = 1.12, se = 0.23, t = 0.54, p = 0.593, 95% CI 0.74 to 1.70). Table 2 presents the results of the analyses adjusted for the confounding effect of the HADS-A baseline score and clustering of patients within sites. These results indicate that the intervention did not significantly reduce the risk of developing a depressive/anxiety disorder compared to usual care. We repeated the analysis on the incidence of depression and anxiety separately as distinct outcomes. Respectively 28 and 19 caregivers in the intervention group and usual care group developed a depressive disorder. Twenty-eight intervention caregivers and 27 usual care caregivers developed an anxiety disorder. Comorbidity was present in 18 of the intervention participants and in 13 of the usual care participants. These differences were again not significant in the distinct analyses (Table 2).

**Table 2:** Incidence of depressive and anxiety disorders over 12 months follow-up.

Primary endpoint	Intervention	Usual care	IRR*	[95% CI]	RD#	[95% CI]
	n (%)	n (%)				
Depressive- or anxiety disorder	38 (39.5)	34 (35.2)	0.98	[0.69; 1.38]	−0.02	[−0.16; 0.13]
Depressive disorder	28 (28.8)	19 (20.2)	1.21	[0.80; 1.84]	0.04	[−0.08; 0.15]
Anxiety disorder	28 (29.3)	27 (28.5)	0.89	[0.51; 1.56]	−0.04	[−0.21; 0.14]

IRR: Incidence rate ratio; CI: Confidence interval; RD: Risk difference.

*intervention versus usual care group, adjusted for HADS-A baseline differences and clustering. An IRR>1 means that over a period of 12 months more caregivers in the intervention group developed a disorder than in the usual care group.

# Risk difference between intervention and usual care group, adjusted for HADS-A baseline differences and clustering.

#### Severity of symptoms

No significant differences on the severity of depressive and anxiety symptoms over time were found between the intervention and usual care group (Table 3). The mean CES-D scores show that the level of depressive symptoms incremented in both groups over the 12 month follow-up period. Anxiety symptoms (HADS-A scores) increased slightly in the usual care group and remained stable in the intervention group.

**Table 3:** Longitudinal changes on continuous outcome measures: depressive symptoms, anxiety symptoms, caregiver burden and health related quality of life.

	Intervention	Usual care	RxT*		
	Adjusted mean[95% CI]	Adjusted mean [95% CI]	Coefficient[95% CI]	t-statistic(df)	p-value
*Primary endpoints*					
**Depressive symptoms (CES-D)**			−1.40 [−3.91; 1.10]	−0.63 (14.0)	0.266
Baseline	11.38 [10.12; 12.64]	11.87 [10.62; 13.12]			
6 months	12.42 [11.07; 13.78]	13.00 [11.63; 14.37]			
12 months	12.89 [11.10; 14.68]	14.78 [13.29; 16.27]			
**Anxiety symptoms (HADS-A)**			−0.55 [−1.59; 0.49]	−1.05 (13.7)	0.296
Baseline	5.61 [5.12; 6.10]	5.32 [4.84; 5.81]			
6 months	5.65 [5.12; 6.18]	5.72 [5.13; 6.31]			
12 months	5.52 [4.74; 6.30]	5.78 [5.12; 6.44]			
*Secondary endpoints*					
**Health related quality of life** **(SF-12)**			−0.75 [−4.81; 3.31]	−0.37 (14.3)	0.715
Baseline	92.15 [89.38; 94.92]	89.65 [86.87; 92.43]			
6 months	91.38 [88.48; 94.27]	89.24 [86.26; 92.21]			
12 months	88.71 [84.67; 92.75]	86.96 [83.20; 90.71]			
**Burden (CRA)**					
Disrupted time			−1.49 [−3.01; 0.02]	−1.99 (14.1)	0.053
Baseline	15.57 [14.67; 16.47]	15.62 [14.72; 16.53]			
6 months	16.21 [15.25; 17.17]	17.24 [16.32; 18.16]			
12 months	15.94 [14.71; 17.17]	17.48 [16.34; 18.63]			
Financial problems			0.50 [−0.28; 1.28)	1.29 (17.9)	0.202
Baseline	6.42 [5.97; 6.87]	6.46 [6.01; 6.91]			
6 months	6.50 [6.01; 7.00]	6.81 [6.32; 7.30]			
12 months	7.24 [6.38; 8.09]	6.78 [6.03; 7.53]			
Lack family support			−0.71 [−1.92; 0.50]	−1.16 (12.7)	0.248
Baseline	13.00 [11.94; 14.07]	12.61 [11.55; 13.67]			
6 months	12.95 [11.74; 14.16]	13.69 [12.47; 14.91]			
12 months	13.20 [11.88; 14.52]	13.51 [12.13; 14.90]			
Health problems			−0.40 [−1.36; 0.57]	−0.81 (18.5)	0.418
Baseline	10.15 [9.53; 10.77]	10.27 [9.66; 10.89]			
6 months	10.41 [9.75; 11.07]	10.71 [10.04; 11.38]			
12 months	10.83 [10.05; 11.61]	11.35 [10.49; 12.20]			
Caregiver's self-esteem			0.62 [−0.56; 1.80]	1.06 (12.8)	0.296
Baseline	27.14 [26.35; 27.94]	26.79 [25.99; 27.59]			
6 months	27.33 [26.47; 28.19]	26.43 [25.45; 27.40]			
12 months	27.56 [26.51; 28.61]	26.59 [25.60; 27.58]			

CES-D: Center for Epidemiologic Studies Depression Scale; HADS-A: Hospital Anxiety and Depression Scales; CRA: Caregiver reaction assessment scale; SF-12: Short Form 12 item version; CI: Confidence interval; R*T: Randomization-by-time interaction.

*The coefficient represents the estimated difference of the scores between the intervention and usual care group over 12 months. Scores were adjusted for HADS-A baseline differences and estimated with multilevel analysis.

### Secondary outcomes

No significant intervention effects were found on any of the dimensions of caregiver burden and on health related quality of life of the caregiver (Table 3).

### Intervention uptake and per protocol analyses

Of those randomized to the intervention group, 91/96 participated in the preparation session, 73/96 attended 1 or 2 family meetings and 44/96 adhered (i.e. completed the preparation session plus 3 or 4 family meetings within 12 months) to the intervention protocol. The 44 adherers did not significantly differ on any of the baseline characteristics compared with the non-adherers, despite that for non-adherers the patient's number of ADL dependencies was slightly higher (difference in mean = −0.56, 95% CI −1.11 to −0.01, t = −2.04 with 93 df, p = 0.044). Reasons for non-adherence were: resistance of family/family conflicts (n = 11), no perceived need for (more) family meetings (n = 10), too burdensome (n = 9), placement in a nursing home or death of the patient (n = 7), practical considerations (n = 5), other reasons (n = 3). Furthermore, seven caregivers finished the intervention, but not within 12 months after enrollment.

Of the 73 caregivers who attended at least one family meeting, 64 completed an evaluation form after their last session. Satisfaction among the participating caregivers was high: 53 (83%) experienced the family meetings as useful, while 8 caregivers experienced no benefits (data on 3 persons were missing/inconclusive). The satisfied caregivers attended more family meetings (difference in mean = 2.3, 95% CI 1.95 to 2.74, t = 11.75 with 94 df, p = 0.000) and experienced a higher lack of family support at baseline than the other intervention caregivers (difference in mean = −1.67, 95% CI −3.27 to −0.08, t = −2.08 with 93 df, p = 0.040). Also, they cared for persons with dementia who were more often diagnosed with Alzheimer Disease (66% versus 44%) and had less ADL dependencies (difference in mean = −0.62, 95% CI −1.21 to −0.04, t = −2.13 with 63.5 df, p = 0.037).

The family meetings lasted on average 73 minutes (range 47–105). The number of family members/friends attending a meeting was 4.4 (range 2–14). In 85%, the demented person was not present at the meetings or only attended a part of the sessions. Family meetings were organized at the office (57%) or at the family's home (43%). The per protocol analyses showed no significant differences between the adherers and usual care group on any of the outcome measures (Table 4).

**Table 4:** Per protocol analyses on the primary and secondary outcome measures.

	IRR*/R*T interaction#	[95% CI]	t-statistic (df)	p-value
*Primary endpoints*				
Depressive- or anxiety disorder, IRR	0.89	[0.55; 1.44]		0.632
Depressive disorder, IRR	1.10	[0.61; 1.97]		0.755
Anxiety disorder, IRR	0.87	[0.48; 1.57]		0.647
Depressive symptoms (CES-D), R*T	−1.30	[−3.91; 1.30]	−0.99 (62.8)	0.325
Anxiety symptoms (HADS-A), R*T	−0.52	[−1.74; 0.70]	−0.84 (14.2)	0.400
*Secondary endpoints*				
Health related quality of life (SF-12) , R*T	0.11	[−4.48; 4.69]	0.05 (24.5)	0.963
Burden (CRA), R*T				
Disrupted time	−1.24	[−2.99; 0.52]	−1.41 (17.5)	0.165
Financial problems	0.29	[−0.65; 1.24]	0.62 (17.0)	0.538
Lack family support	−0.88	[−2.44; 0.67]	−1.13 (16.9)	0.262
Health problems	−0.38	[−1.67; 0.91]	−0.59 (15.6)	0.554
Caregiver's self-esteem	0.14	[−1.17; 1.45]	0.21 (15.7)	0.834

IRR: Incidence rate ratio; CI: Confidence interval; CES-D: Center for Epidemiologic Studies Depression Scale; HADS-A: Hospital Anxiety and Depression Scales; CRA: Caregiver reaction assessment scale; SF-12 Short Form 12 item version; CI: Confidence interval; R*T: Randomization-by-time interaction.

*intervention versus usual care group, adjusted for HADS-A baseline differences and clustering. An IRR >1 means that over a period of 12 months more caregivers in the intervention group developed a disorder than in the usual care group.

# The randomization-by-time interaction coefficient represents the estimated difference of the scores between the intervention and usual care group over 12 months. Scores were adjusted for HADS-A baseline differences and estimated with multilevel analysis.

### Use of health care and supportive services

For 92/96 caregivers in the usual care group and 89/96 intervention caregivers data on the health care use and supportive services were available. We found that 52 caregivers in the usual care group received additional counseling from a psychologist, case manager or social worker and 51 caregivers in the intervention group received such counseling (χ^2^ = 0.011, df = 1, p = 0.915). Twenty caregivers in the usual care group reported participation in a support group versus 19 caregivers in the intervention group (χ^2^ = 0.004, df = 1, p = 0.949).

### Ancillary analyses

We pre-specified several variables which we assumed as potential effect modifiers: caring for a more severe demented patient (MMSE≤median of 22) and caregiver gender. Furthermore, we explored whether caregivers having a dementia case manager (yes/no), caregivers with increased initial depressive (CES-D≥16) or anxious (HADS-A≥8) symptoms, and caregivers with a lack of family support (CRA subscale<median of 12) would benefit (more) from the intervention. We did not find any evidence for effect modification.

During the study period, 24 patients were institutionalized, 3 died and 6 died after placement. Sensitivity analyses were carried out using only caregivers where the patient survived and lived at home during the entire study. These analyses did not demonstrate significant effects of family meetings on any of the primary and secondary outcomes.

## Discussion

### Interpretation

This study was the first to examine the preventive effects of a structured family meetings intervention for family caregivers of dementia patients. The intervention did not prevent the onset of depression or anxiety disorders, nor reduced symptom levels and caregiver burden. The incidence of depression and anxiety disorders was equally substantial in both groups of relatively young caregivers. Within 12 months, almost 40% of the caregivers developed a mental disorder according to diagnostic criteria. This incidence is far higher than in the cohort study of Joling et al among spouses of dementia patients [3] and than found in ‘general’ elderly cohorts and emphasizes the vulnerability of this target group [28]–[30].

### Overall evidence

Counseling and support interventions that include family meetings were investigated by Mittelman et al., who found small to moderate but significant effects that lasted more than 3 years on depressive symptomatology in caregivers [16], [17]. Subsequently, a multinational study carried out a similar intervention among spouses of persons with Alzheimer disease taking donepezil and also reported a small but significant reduction in depression scores [31].

The lack of effects in our study may be due to the fact that the intervention did not include all components of the multi component NYU Caregiver Intervention. Perhaps the intervention lacked the same time condensed delivery to have an impact on the outcomes compared to the original protocol in which the counseling sessions were delivered within 4 months after the intake assessment, and followed with ad hoc counseling from the same counselor. However, in the Netherlands, there are many supportive services available to caregivers and the provided standard care is already quite intensive in a substantial number of cases. This relatively high level of usual care may have resulted in a limited contrast between the intervention and usual care group and might be a reason for the lack of effects. The non-significant findings are in accordance with recent meta-analytic reviews which demonstrated modest evidence for significant effects of psychosocial interventions [11], [12]. Combined intervention programs for both the caregiver and person with dementia were often effective in delaying long stay care admittance, but to a lesser extent in improving caregivers' mental health [32]. The most successful interventions used a psycho-educational or psychotherapeutic approach, addressed multiple stressors, were better adapted to the individual needs of the caregivers and provided a higher amount and intensity of support [13], [33]. Most positive effects were found in the subgroup of female caregivers and caregivers caring for people with a dementia diagnosis ‘not otherwise specified’[34].

The lack of effectiveness in this study may also be due to the fact that the participants' compliance with the intervention was not optimal. Several strategies were taken to maintain a high level of participation. The sessions were scheduled at the convenience of the family as much as possible and the counselors provided the family meetings in the caregivers' homes if they were unable to leave the patients. Nevertheless, about one-quarter of the intervention caregivers participated in no family meeting at all and about half of them completed the majority of the sessions. However, this could also mean that this type of intervention is not what these relatively young caregivers think that they need.

Another possible explanation for the lack of protective effect in our study may be that the timing of the intervention was not appropriate. In contrast with some other studies evaluating family meetings, we excluded caregivers with major depression or anxiety at intake to be able to evaluate preventive effects on onset of such a disorder. Almost 30% had baseline CES-D scores above the cutoff, indicative of clinically relevant symptoms of depression. This was lower than the 43% of the caregivers who had scores at baseline above the cutoff on the Geriatric Depression Scale indicating possible clinical depression at baseline in the NYU Caregiver Intervention study. Current research in noncaregiver populations suggests that prevention is most likely to be effective when targeting people with increased symptoms levels [8]–[10]. Indicated prevention might also be a promising research strategy for the caregiver population and perhaps family meetings interventions would be most useful to caregivers with more severe symptom levels at baseline. Furthermore, a recent study presenting an overview of successful psychosocial interventions in subgroups of caregivers of people with dementia, showed that the presence of mental health problems was frequently related to positive intervention effects for caregivers [34]. However, evaluation of possible effect modification by increased initial depressive or anxiety symptom severity in our study did not show more benefit of the intervention over usual care.

Unlike the NYU Caregiver Intervention-model, the two individual and four family counseling sessions were conducted with a frequency of every 2 to 3 months instead of offering all sessions in the first four months after enrollment. This might indicate that, in order to affect change in support for the primary caregiver, family meetings should be conducted with greater frequency in a shorter period of time. A pilot with some families prior to the study was an important reason to choose a larger time span between the session. Also, from the evaluation forms filled in by the intervention caregivers who participated in the trial, we found that most of them were satisfied with the amount of sessions they received. Perhaps caregivers did prefer a relatively low frequency, because a majority of them also used other supportive services. This shows an important dilemma of following the participant's preferences on the one hand, and on the other hand stick to the current evidence that interventions tend to have stronger effects if they involve more frequent interactions [13], [35].

It is also possible that effects might become significant in the long term. In the NYU Caregiver Intervention study, changes in depressive symptoms were significant approximately 6 months after the last counseling session (approximately 10 months after enrollment). The 12 months follow up period after enrollment in our study might be too short to detect significant effects, but this is not very likely in view of the current findings.

According to the random selection of audiotapes that we listened to, the standardized forms that were completed by the counselors after each session and the contact between the research team and counselors during the intervention period, the counselors mostly carried out the intervention as instructed by the manual and during the training organized prior to the study. Furthermore, all counselors were uniformly trained, used a structured manual and may be assumed to be adequately qualified to lead the family meetings. Therefore, we might assume that the actual quality of the intervention was no reason for the lack of effects in our study. Considering the randomized design, the adjustment for baseline differences associated with the outcomes, the relatively large number of subjects, the low drop out rate (from follow-up) and the adjustment for possible clustering effects, it seems unlikely that the non-significant results were due to methodological flaws.

### Generalisability

We performed a randomized controlled trial with only a few exclusion criteria. A substantial number of caregivers invited for the study were not willing to participate, mainly due to a lack of perceived need for this intervention. Although this limits the feasibility of this intervention for many caregivers, the results reflect the effectiveness in usual routine care for caregivers who were interested in this intervention. Caregivers in our sample were predominantly women caring for a –relatively young- demented spouse, while according to population based studies about half of the demented patients in this age group are women [36], [37]. A care setting based study like our study starts with people who asked for care or a diagnostic assessment and therefore the gender distribution could differ from the one reported in population based studies. Participants in our study did not differ significantly from the persons who declined participation with regard to gender, patient-carer relation, and the service they were recruitment from, and therefore, our sample seems to be representative for the population that receives treatment or diagnostic assessment at these services.

### Conclusions

This study did not show any preventive effects of family meetings compared with usual care. The substantial number of invited caregivers unwilling to participate and poor intervention uptake indicates that family meetings are only acceptable for some of the caregiver population. This also reflects the diversity among caregivers in their need for different types and intensity of support. Although this intervention could not prevent caregivers from developing depression and anxiety, certain family caregivers may feel supported and satisfied about this intervention. Although, from our results no subgroups emerge for whom the intervention could work for, it can be hypothesized that caregivers with certain characteristics we did not take into account (like coping style) might benefit more from family meetings. The finding that caregivers were more satisfied about the family meetings when experiencing a higher lack of family support prior to the intervention might indicate that family meetings better fulfill the needs of the caregiver when a certain lack of family support is experienced.

Researchers might consider studying the effects of organizing family meetings for specific subgroups of caregivers or with a therapeutic purpose. Further research should determine whether family meetings alone, might then be more beneficial if delivered more intensively over a shorter period of time or whether the intervention's effectiveness may be derived from its multicomponent nature. Other studies should be conducted to evaluate the transportability of evidence-based interventions such as the NYUCI to cultures in which usual care provide substantial support for family caregivers.

## Supporting Information

Protocol S1 **Trial Protocol.**
(PDF)

Checklist S1 **CONSORT Checklist.**
(PDF)
