# Supplementary material for: Does a Family Meetings Intervention Prevent Depression and Anxiety in Family Caregivers of Dementia Patients? A Randomized Trial
Source: PLoS One. 2012 Jan 27;7(1):e30936. doi: 10.1371/journal.pone.0030936 (PMC3267736; doi:10.1371/journal.pone.0030936)
Supplement: Protocol S1 — Trial Protocol. (PDF) [file pone.0030936.s001.pdf]

RESEARCH PROTOCOL

FAmily MEetings in Memory Clinics (FAME)

PROTOCOL Family Meetings in Memory clinics: Prevention of developing Anxiety and Depressive Disorders in primary informal caregivers of demented patients

**Short title**	**Family meetings in Memory Clinics**
**Version**	**4**
**Date**	**March 2009**
**Coordinating investigator/project leader**	***Dr. HPJ van Hout***<br>***Van der Boechorststraat 7***<br>***1081 BT Amsterdam***<br>***hpj.vanhout@vumc.nl***
**Principal investigator(s) (in Dutch: hoofdonderzoeker/uitvoerder)**	***Drs. KJ Joling***<br>***VUmc - EMGO***<br>***Afdeling Huisartsgeneeskunde***<br>***Boechorststraat 7***<br>***1081 BT Amsterdam***
**Sponsor (in Dutch: verrichter/opdrachtgever)**	***ZonMw nr 6230.0040***<br>***Postbus 93 245***<br>***2509 AE Den Haag***
**Independent physician(s)**	***B.M.J. Uitdehaag, neuroloog***

PROTOCOL SIGNATURE SHEET

**Name**	**Signature**	**Date**
**Sponsor or legal representative:**<br>**For non-commercial research,**<br>**Head of Department:**<br>***Prof dr. H. van der Horst***		27/3/'09
**Coordinating Investigator/Project leader/Principal Investigator:**<br>***Dr. HPJ van Hout***<br><br>***Drs. KJ Joling***		27-03-'09<br><br>27-03-'09

TABLE OF CONTENTS

SUMMARY 5
1 INTRODUCTION AND RATIONALE 6
1.1 Motivation 6
1.2 Scientific and social relevance 6
1.3 Innovation 8
2 OBJECTIVES 9
2.1 Primary Objective 9
2.2 Secondary Objective 9
3 STUDY DESIGN 9
3.1 Design 9
3.2 Duration and Planning 9
3.3 Setting 9
4 STUDY POPULATION 11
4.1 Population (base) 11
4.2 Inclusion criteria 11
4.3 Exclusion criteria 11
4.4 Sample size calculation 11
5 INTERVENTION 12
5.1 Description 12
5.2 Intervention stages 12
5.3 Training 13
5.4 Usual care 13
6 Methods 14
6.1 Study parameters/endpoints 14
6.2 Informed consent 15
6.3 Study procedures 15
6.4 Randomisation, blinding and treatment allocation 15
6.5 Statistical Analysis 15
6.6 Withdrawal of individual subjects 15
7 SAFETY REPORTING 17
7.1 Section 10 WMO event 17
7.2 Adverse and serious adverse events 17
8 ETHICAL CONSIDERATIONS 18
8.1 Regulation statement 18
8.2 Recruitment and consent 18
8.3 Objection by minors or incapacitated subjects 18
8.4 Benefits and risks assessment, group relatedness 18
8.5 Compensation for injury 18
9 ADMINISTRATIVE ASPECTS AND PUBLICATION 19
9.1 Handling and storage of data and documents 19
9.2 Amendments 19
9.3 Annual progress report 19
9.4 End of study report 19
9.5 Public disclosure and publication policy 19
REFERENCES 20

SUMMARY

Rationale
The growing group of family caregivers of dementia patients has a highly increased risk of developing depressive and anxiety disorders. An American landmark study reported substantial beneficial effects of family meetings on depression in family caregivers as well as on delay of institutionalisation of patients. Effects are not replicated in other countries yet. When effective, family meetings can be an important addition to the current care services in the Netherlands.

Objective
To investigate the effectiveness of family meetings on prevention of anxiety and depressive disorders (DSM IV) and symptom levels of primary family caregivers of patients with dementia. To perform an economic evaluation alongside the trial.

Study design
Randomised controlled clinical trial comparing structured family meetings versus usual care with significant others of 172 primary family caregivers of community dwelling demented patients recruited in memory clinics with a follow up of 24 months.

Study population
Dyads of diagnosed dementia patients and their central informal family caregiver. The unit of analysis is the caregiver.

Intervention
Four meetings with family and close friends will be organized and run by a trained counsellor according to a manual (Mittelman 2003). The aim is to offer psycho-education, increase problem-solving skills and mobilize the naturally existing social network of patient by sharing support tasks of network members.

Main study parameters/endpoints
Incidence of anxiety and depressive disorders (DSM IV minor, major depression, generalised anxiety, panic disorder, phobic disorders) assessed with the Mini-International Neuropsychiatric Interview (MINI). The *dimensional* or severity measure of anxiety and depression *symptoms* is derived by validated self report instruments: the Centre for Epidemiologic Studies Depression Scale (CES-D) and Geriatric Depression Scale (GDS-5) for depression and the anxiety subscale of the Hospital Anxiety and Depression scales (HADS) for anxiety. Additional questionnaires are used to explore profiles of caregivers who are best helped by the intervention.

Nature and extent of the burden and risks associated with participation, benefit and group relatedness
The burden of the intervention will be minimal for the dementia patients and their family caregivers. A possible burden can be created by the number of questionnaires which will be taken to assess the study parameters. Furthermore, emotional discomfort and family conflicts can exist as a consequence of participation in the intervention. This possible burden will be in proportion to the potential value of the research.

1 INTRODUCTION AND RATIONALE

1.1 Motivation

Our hypothesis is that affective disorders (i.e. depressive or anxiety disorders) of dementia caregivers who are already at risk for such disorders are largely preventable. Systematic reviews and meta-analyses show that information and support alone is helpful but only address the psychological needs of caregivers modestly at best (Sorensen 2002, Brodaty 2003). Programs that demonstrate beneficial effects on affective disorders involved both patients and their families, are more intensive and modified to caregivers needs (Brodaty 2003, Acton 2002). As demonstrated in the landmark studies of Mittelman et al., family meetings, designed to mobilize support of naturally existing family networks, appear to be among the most powerful psychosocial interventions to reduce depression in caregivers (Whitlatch 1991, Mittelman 1995, 1996, 2004). However, these studies have not been replicated elsewhere. Family counselling can maximise the positive contributions of each member to caregiving, prevent one member from carrying the entire weight of the caregiving role, improve the caregiver's understanding of how to ask for help, what kind of help is reasonable to expect from family members, and how to accept help, and reduce family conflict. In addition, these programs resulted in postponement of nursing home placement of patients, while maintaining a comparable or better quality of life of the caregivers.
Our study has several modifications compared to the work of Mittelman. Mittelman's studies were performed among spouse caregivers of patients with Alzheimer type dementia only. The question remains whether the intervention is robust when a group of caregivers is included that is more heterogeneous. Spouses, children, neighbours, and friends may play a caring role. The effects of family meetings on child-caregivers and in carers of non-Alzheimer type dementia patients are not studied yet. Also, possible preventive effects on anxiety, a frequent co-morbid condition in depressed caregivers, were not evaluated yet. Mittelman's intervention consisted not only of family meetings but also of individual counselling and support groups for caregivers, as well as the possibility of telephone counselling for caregivers and other family members as needed. Much of the beneficial effects were attributed specifically to the family meetings. However, it remains to be seen whether family meetings alone will demonstrate comparable preventive effects.
Despite its great potential, Dutch health care professionals rarely organise family meetings. In this age of shared care, memory clinics seem especially well suited to organize family meetings: a) memory clinics are enthusiastic to participate, b) they already have the necessary expertise, the personal and/or are willing to learn; c) family meetings are a logical next step for them: their usual practice traditionally focused on the medical aspects, the diagnostics of memory problems and medication management since anti-Alzheimer medication has become available (Verhey 2005). Family meetings also have a social diagnostic dimension (assessment of support); d) The number of Dutch memory clinics has increased rapidly to about 40 currently. They already diagnose about 25% of all dementia patients (coming from 5% in 1998) and this percentage is expected to increase further to about 50% over the next years (Verhey 2005). Nevertheless, family meetings are rare and are not performed in a systematic way with follow up meetings.

1.2 Scientific and social relevance

Impact

Many studies demonstrated the enormous impact of depressive and anxiety disorders and symptoms on the quality of life. Depressive disorders rank second in the World Health

Organisation's list of disorders with the most quality adjusted life years lost (Murray 1997). Anxiety disorders are strongly associated with depressive disorders and represent a comparable burden (Beekman 2000).
Family caregivers have an extremely high risk of becoming depressed or anxious as well as a higher mortality risk, and family caregivers of persons with dementia even more so than other caregivers(Schulz 1999). Dementia is strongly associated with aging: the prevalence figures double every five years of increase in age (Hofman 1991). In our rapidly aging population the projection of dementia prevalence in 2050 will have doubled (GR2002).
Despite a wide range of useful services that can help patients and relief caregivers, the burden of caregiving remains very high (Schulz 1999). Long term institutionalisation of persons with dementia is extremely costly. The mental health of the family caregiver is often decisive in the timing of nursing home placement. Any intervention that can relieve the burden of caregiving and (thereby) prevent mental health problems of caregivers is important. In addition, if such efforts can delay nursing home placement they are likely to be extremely cost-effective.
The family can be both an invaluable aid to a caregiver and a source of stress. Three quarters of dementia caregivers reported conflict with family members who were not seen as doing their share of caregiving and 73% of the dementia caregivers reported mental and physical health problems compared to 50% respectively 22% of non-dementia caregivers (AARP 1997). In general, 76% of caregivers care with satisfaction; only 13% feels obligated to do so (SCP 2003). These caregivers should be enabled to provide care without becoming a patient themselves.

Target group

Family members of persons with dementia, often at great personal cost, provide much of the care for older adults with dementia in the community (Cohen 1999). Family caregivers of dementia patients have an extremely high risk developing affective disorders such as major depression and anxiety disorder, with women and spouses at even higher risk (Cuijpers 2005, Schulz 1999). This risk persists over many years of caregiving and even after caregiving ends with the death of a care recipient (Schulz 1999, Robinson-Whelen 2001). Generic preventive screening is not advocated (Cuijpers 2002). However, especially among those who already have symptoms of distress or burden, indicated prevention may be the road forward. An additional advantage of prevention of affective disorders of caregiver is that the period until institutionalisation may be prolonged while maintaining a comparable or better quality of life of the caregivers.
Family caregivers generally prefer to avoid placing their elderly relatives in residential care, and spouse caregivers are more reluctant than other relatives to do so. Family caregivers willingness to care has not declined over the last decade as might be expected due to loosening of family ties (SCP 2003, STG 1990). Professional services should at least offer as much effective evidence based outpatient support as possible.

Prevalence and incidence

The month prevalence of depressive disorders among caregivers varied between 15-32% in three representative community samples, the 12 months incidence of depressive disorders was 48% as reported in one representative sample (Cuijpers 2005, Ballard 1996). Data on anxiety disorders in dementia caregivers are scarce but suggest that 1 in 3 caregivers suffers from an anxiety disorder (Akkerman 2004). Depressive and anxiety symptoms are even more prevalent and affect between half to three-quarter of all caregivers (Cuijpers 2005, Ballard 1996, AARP 1997).

The estimated prevalence rate of dementia among community dwelling elderly aged 65 to 95 is 6.6% (Hofman 1991). In the Netherlands, currently 1 in 93 persons suffer from dementia (about 200.000 patients). Two third lives at home and a family member cares for most of them. A cure for dementia is not within reach in the near future. Over the next years, the number of demented elderly will increase substantially and so will be the numbers of family caregivers caring for a demented person. In 2050, as a result of aging populations, about 1 in 44 persons will be demented (GR 2002). Consequently, the number of informal caregivers, at risk for psychological morbidity, will have doubled as well by that time.
Recent reports by national advisory bodies
Both nationally and internationally there is great concern regarding the enormous burden and costs of dementia related care (GR 2002, RIVM site). The Dutch national health council identified an urgent under capacity of residential facilities for demented persons, which cannot be solved in short notice.

Theory
Psychosocial interventions such as empowering families' support system by counselling in family meetings, are based on the theory of the stress process that suggests that by improving certain moderators, social support and mastery over the caregiving task, it is possible to create buffers against the burden of caregiving on outcomes such as depression, anxiety, an disability to continue provide care outside of an institution (Pearlin 1978).

1.3 Innovation

The effectiveness of using only the family counselling component of Mittelman's intervention has not been tested. Effects of family meetings were not replicated in other countries yet. In addition, the earlier study was done with spouse caregivers of Alzheimer type dementia patients only and measured effects on depression but not on anxiety. In our study we also include other family caregivers relations as well as non-alzheimer type dementias and measure both depression and anxiety.
Finally, economic evaluations of this intervention were not reported yet. When (cost)-effective, it can be an important addition to the current care services in the Netherlands.
Are there similar studies underway: no similar studies are running currently.

2 OBJECTIVES

2.1 Primary Objective

To investigate the effectiveness of family meetings on the subsequent two year prevention of development of anxiety and depressive disorders (DSM IV) and symptom levels of primary family caregivers of patients with dementia.

2.2 Secondary Objective

An economic evaluation will be performed alongside the study to estimate the cost-effectiveness.

3 STUDY DESIGN

3.1 Design

Randomised controlled indicated prevention trial.
After selection and baseline assessment, study subjects are randomised to one of two groups; the intervention group receives usual care plus family meetings, the control group receives usual care only. Participants are free to seek additional assistance and support elsewhere at any time throughout 24 months follow-up. The study subjects are recruited through several memory clinics and specialized mental health care institutions.

3.2 Duration and Planning

Total 62 months: 10 months preparation; 22 months inclusion and baseline measurements, 24 months follow up, 6 months analyses and reports.

Planning

Jan 2007 - Nov 2007	Preparation:10 months
Nov 2007 - Sept 2009	Inclusion and baseline measurement: 22 months
Sept 2009 - Sept 2011	Follow-up: 24 months (measurements at 6, 12, 18 and 24 months)
Sept 2011 – Dec 2012	Analysis and reports: 6 months

Time schedule

	jan	febr	march	april	may	june	july	aug	sept	oct	nov	dec
2007	preparation										inclusion	
2008	inclusion											
2009	inclusion								follow up			
2010	follow up											
2011	follow up								analyses			
2012	Analyses											

3.3 Setting

Several memory clinics and specialized mental health care institutions.

The flow chart gives an overview of the procedures that subjects will undergo in the course of research.

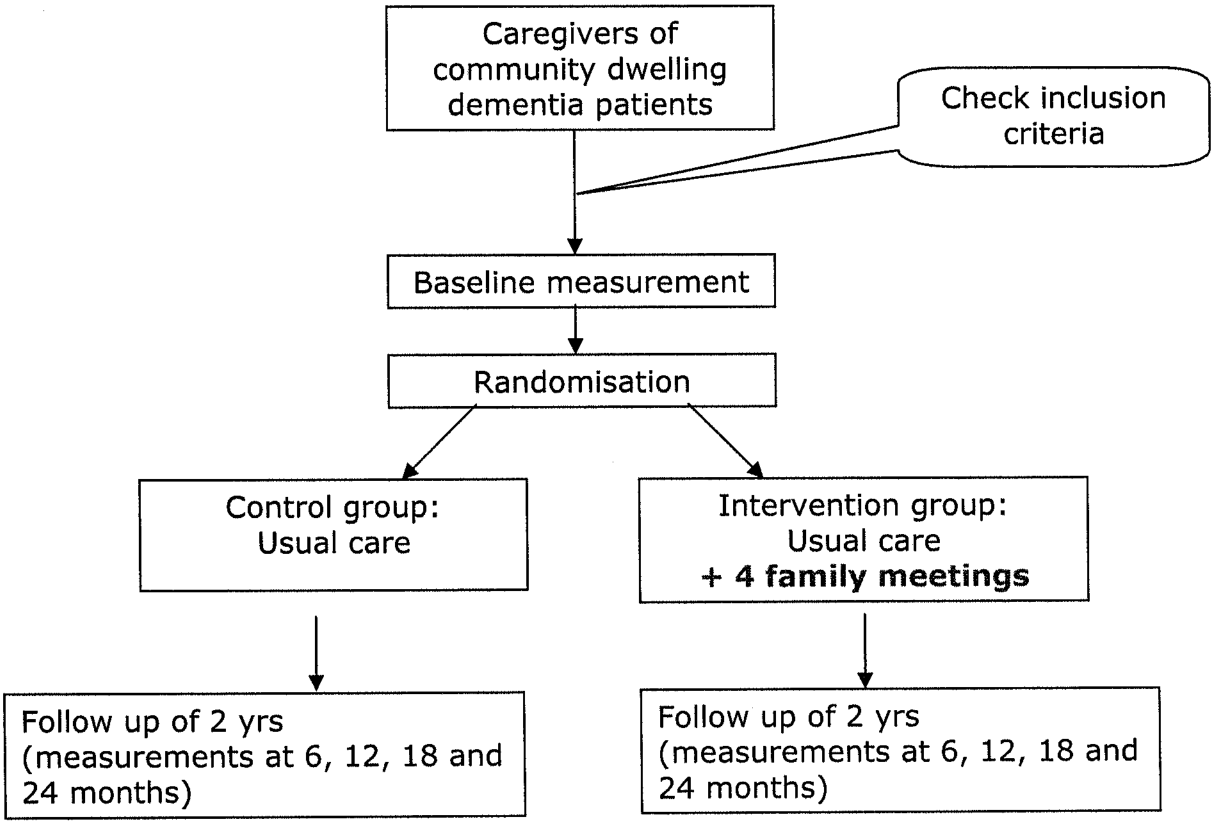

4 STUDY POPULATION

4.1 Population (base)

Caregivers and patients will be drawn from memory clinics and several memory clinics and specialized mental health care institutions with a combined annual turnover of more than 750 diagnosed dementia patients. Therefore, even bearing Lasagna's law (researchers and clinicians invariably overestimate the number of patients available for study participation) in mind, we feel confident that we can achieve our targeted number of caregiver-patients dyads within 22 months. From our previous studies, we learned that an inclusion period of 18 months is sufficient to include the required number of pairs.

4.2 Inclusion criteria

- Family caregiver who takes primary responsibility for the informal care of a community dwelling patient with a clinical diagnosis of dementia.
- Both caregiver and patient have sufficient language proficiency in Dutch for adequate participation in meetings, interviews and tests.
- Written informed consent from both patient and caregiver is obtained. In case of mental incompetence of a patient the family caregiver will sign the consent for the patient.

4.3 Exclusion criteria

- Severe somatic or psychiatric co-morbidity of either caregiver or patient, which will significantly impair cooperation to the program.
- Either caregiver or patient participates in other intervention studies at inclusion or during the study.
- Caregivers having a depressive or anxiety disorder.
- Scheduled to move a patient to a nursing home.

4.4 Sample size calculation

Sample size calculations are based on the expected effects of the intervention on the main outcome measures, incidence of affective disorders and increasing time to incident problems. Incidence estimates are derived from the literature and other studies performed by our group (Prevention of anxiety and depression in late life). Effect estimates are based on the primary studies of Mittelman (1996, 2004). We estimate the yearly incidence of affective disorders among caregiver at risk conservatively at 30% (Cuijpers 2005). We aim to reduce the incidence to 10% of new cases a year. With an alpha of 0.05, power of 80%, 73 persons per group are then needed. Assuming 15% loss to follow up we need to recruit 2x73x100/85=172 pairs of dementia patients and family caregivers (Statpower software). This number will be sufficient as well to measure a moderately strong reduction (Cohen's D>0.5) in number of depressive or anxiety symptoms.

5 INTERVENTION

5.1 Description

The purpose of family counseling is to help family members provide emotional and instrumental support to the primary caregiver, the family member who carries the greatest weight of the caregiving, and to help all family members adapt to the changes that the demented process imposes on them.
The four family counseling sessions will follow the manual (Mittelman 2003). Four family meetings are scheduled within 8 months and run by a counselor with an advanced degree in nursing, social work or allied professions. Counselors receive a training based on the manual and train their skills prior to the study. One counselor is assigned to one caregiver to establish an ongoing relationship with someone who is familiar with the situation. The aims of the family meetings are: 1) to educate families about dementia consequences and resource information for care support; 2) to mobilize naturally existing family networks of patient and primary caregiver in order to improve emotional and instrumental support; 3) to teach problem solving and techniques for troublesome patient behavior that can be used after family counseling ends. The content of the sessions is also determined by the needs of each caregiver. For example, learning techniques, for management of troublesome behavior, promoting communication between family members. All sessions are audio-taped for supervision, intervision and detailed analysis.

5.2 Intervention stages

Preparation
The first family session is prepared after the disclosure session with the patient and the caregiver. A follow up contact is planned soon after the disclosure session. The counsellor introduces the idea of seeking help from family members or friends and explains why this is beneficial. The caregiver defines and invites the family. A family can comprise good friends or neighbours as well. Counsellor and caregivers decide whether the patient attends the meetings as well. The counsellor recognizes the degree of openness in a family, the communication style, assesses the knowledge of dementia and is aware of individual coping styles.

Initial meeting
Explanation of the purpose of the meetings, the protocol, scope of services that can be provided, ground rules and the counsellor's role; Introduction of all persons and expression of their perspectives on the situation; Clarification of issues and getting a consensus on which to address; Identification of objectives and helping family to allocate the tasks; Summary of observations and making plans for the next session.

Follow up
Review of the previous session, previous commitments, task assignments, discussion of progress of carrying out tasks, identifying issues for current consideration. During the session it will become clear that some issues are resolved while others have taken a higher priority. In addition, new issues, problems, and conflicts will emerge. These are discussed in the same way as the original issues were in the first session. The counsellor concludes by discussing the date and time of the next session and who will attend. If the next meeting will be the last family session, the family should be reminded of that fact so that it does not come as a surprise and the can prepare for termination.

Final session
Acknowledge that this is the last session, give a summary of the observations about the progress that has been made and the goals that remain to be achieved; relate new issues to previously solved issues.

Follow-up
When needed additional ad hoc counseling for the family caregiver can be offered. Expectedly this will happen extremely incidentally.

5.3 Training

An English Manual on how to do family meetings is available (Mittelman 2003). At the VUmc we have made a translation of the most important parts. Personnel of the memory clinics are trained according to this manual. Also Dr Vernooij-Dassen have already gained some experience with family meetings and will supervise ongoing meetings. Every site will pilot some meetings before the effect study will start. The VUmc site has already begun to pilot family meetings. So far we have met enthusiastic reactions from families.

5.4 Usual care

The diagnostic assessments at memory clinics lead to a disclosure meeting with the patient and in most cases the primary family caregiver. Patients diagnosed with dementia and their accompanying family members are informed about the facilities that can help caring for the patients. Helpful leaflets, addresses, phone number and web sites are provided. Next, most patients are referred back to their General Practitioner. About a quarter of Alzheimer patients is prescribed anti-Alzheimer medication, and in such cases patient and family caregiver visit the memory clinic regularly for medication control visits. Family meetings are organised rarely and never in a structured way and with follow-ups. Moreover they tend to focus on providing clinical information and not on increasing family support.

6 METHODS

6.1 Study parameters/endpoints

Main study parameter/endpoint

- Incidence of major depression and anxiety disorders (i.e. generalised anxiety and panic) as defined according to DSM-IV criteria (APA 1994) and assessed with the Mini International Neuropsychiatric Interview (MINI) (Sheehan 1998). The MINI is used as 3 month prevalence measure.
- The *dimensional* or severity measure of anxiety and depression *symptoms* is derived by validated self report instruments: the Centre for Epidemiologic Studies Depression Scale (CES-D) (Radloff 1977) and Geriatric Depression Scale (GDS-5) (Hoyl 1999) for depression and the anxiety scale of the Hospital Anxiety and Depression scales (HADS) for anxiety (Zigmond 1983; Spinhoven 1997).

Secondary study parameters/endpoints

Caregiver
- Caregiver burden with the Short Sense of Competence Questionnaire (SSCQ) (Vernooij-Dassen 1999) and the Caregiver Reaction Assessment (CRA) (Given 1992)
- Quality of life with the Short Form 12 item version (Ware 1995)
- Quality adjusted life years with the SF-6D (SF-12)

Patients
- Depressive symptoms in patients (NPI)
- Quality of life with the Dementia Quality of Life Instrument (DQoL)

Other
- Days until institutionalization
- Resource utilization of patient and carer will be measured from a societal perspective with cost diaries (filled out by the carer) which include both direct (hospital visits) and indirect costs (travel time) and both within and outside (loss labour days) the health care.

Potential confounders/modifiers
- Demographics patient and caregiver (age, sex, SES)
- Family relation patient-caregiver
- Duration and hours of caregiving
- Type of caregiving (instrumental, 24 hours supervision, travel)
- Social support of caregiver with a Social Support Assessment based on the Caregiver Assessment Battery used in the studies of Mittelman (Mittelman 2006).
- Loneliness with the Jong-Gierveld loneliness scale (de Jong-Gierveld 1985)
- (Instrumental) Activities of daily living of the patiënt (Lawton 1969; Katz 1963)
- Type of dementia
- Cognition of patients with the Mini Mental Status Examination (MMSE, Folstein 1975)
- Behavioural symptoms: Neuropsychiatric Inventory (NPI) (Cummings 1998)
- Medical co-morbidity and treatment (e.g. anti-depressants, benzodiazepines, anti-Alzheimer drugs, dementia type)

- Formal care support (e.g. respite care, meeting centre, elderly welfare advisor, Alzheimer café, home nursing)

6.2 Informed consent

Written informed consent from both patient and caregiver is obtained. In case of mental incompetence of a patient the legal representative will sign the consent for the patient. If there is no legal representative or curator the family caregiver will sign the consent for the patient (only spouses, children (-in-law) and brothers/sisters of the patient). We will emphasize that the intervention focuses mainly on the informal caregiver. The dementia patient will always receive the care he or she needs and the family meetings will be an extra service to support the primary caregiver. The data of the patient are obtained to measure whether the patients quality of live improves if his/her informal caregiver gets more support. The data of the patient is also needed to correct for potential confounders and modifiers.

6.3 Study procedures

After informed consent both patient and primary caregiver have to fill in several questionnaires (patients will be interviewed depending on their capabilities). The measurements at baseline and follow up (at 6, 12, 18 and 24 months after baseline) are done by independent and blinded assessors.

6.4 Randomisation, blinding and treatment allocation

Pairs are randomized after baseline measurement. We will be able to detect and monitor selection bias as eligibility of caregivers will be routinely tested during the diagnostic work up. Baseline and follow up data are gathered by independent and blinded interviewers.
After randomisation, participants included in the intervention will follow the different stages (described in paragraph 5.2)

6.5 Statistical Analysis

Intervention effects will be analysed with survival analysis (Cox Proportional Models) for the time to incident affective disorder and repeated measurement analyses for number of symptoms. Both techniques can adjust for possible baseline imbalance, and potential modifiers (e.g. gender caregiver, relation with patient, patient centre). Dropout and loss to follow up will be described.

6.6 Withdrawal of individual subjects

Subjects can leave the study at any time for any reason if they wish to do so without any consequences. The investigator can decide to withdraw a subject from the study for urgent medical reasons.

Follow-up of subjects withdrawn from treatment
Data will be primarily analysed according to the Intention to treat principle, i.e. including all participants with valid data, regardless of whether they did or did not receive the intervention. Subsequently, the results of the intention to treat analysis will be compared with the results of the 'on treatment' analysis, to assess whether protocol violations have caused bias. Participants with documented deviations from the protocol (i.e. participants who did not receive the entire intervention or participants in either the intervention or the control group with incomplete

follow-up data) will be excluded from the on treatment analysis. Comparability between the intervention and usual care groups will be assessed at baseline to check differences.

7 SAFETY REPORTING

7.1 Section 10 WMO event

In accordance to section 10, subsection 1, of the WMO, the investigator will inform the participants and the METC-VUmc if anything occurs, on the basis of which it appears that the disadvantages of participation may be significantly greater than was foreseen in the research proposal. The study will be suspended pending further review by the METC-VUmc, except insofar as suspension would jeopardise the subjects' health. The investigator will take care that all subjects are kept informed.

7.2 Adverse and serious adverse events

It is very unlikely that the intervention will cause serious adverse events as it concerns a psychosocial intervention. This was demonstrated in earlier studies which organized family meetings in a similar way (Mittelman 2003). The only imaginable side-effect may be that the patient is confronted in a more intensive way with the nature of his or her mental condition. However, this may also be the reason why this intervention works. The pilot which the VUmc site already started has given positive reactions from the participating families as well. When serious adverse events will appear, it will be reported to the METC-VUmc according to the requirements of that METC.

Follow-up of adverse events
All adverse events will be followed until they have abated, or until a stable situation has been reached. Depending on the event, follow up may require additional tests or medical procedures as indicated, and/or referral to the general physician or a medical specialist.

8 ETHICAL CONSIDERATIONS

8.1 Regulation statement

The study will be conducted according to the principles of the Declaration of Helsinki (version 2004) and in accordance with the Medical Research Involving Human Subjects Act (WMO) and other guidelines, regulations and Acts.

8.2 Recruitment and consent

Patients and their primary informal caregiver will be recruited by the participating memory clinics. In every memory clinic a coordinator will be appointed who is responsible for the inclusion procedure. Eligible pairs will be invited for the study after the disclosure session by the responsible clinician. Participants are only allowed to enter the study after signed informed consent. This procedure is described in paragraph 6.2.

8.3 Objection by minors or incapacitated subjects

This psychosocial intervention focuses mainly on the informal caregiver. Only capacitated informal caregivers will be included in this study. The primary caregivers of incompetent patients will decide whether the patient will enter the study and participate in the family meetings. They will also help to complete the patient questionnaires which assess the study parameters.

8.4 Benefits and risks assessment, group relatedness

The primary informal caregiver will be interviewed to assess the incidence of anxiety and depressive disorders in the study population. Patients will be interviewed to measure their quality of life and cognitive functioning. Emotions and conflicts between family members of the dementia patient can be a possible discomfort as a consequence of the participation in family meetings. In our opinion, this possible burden will be in proportion to the potential value of the research. There are many indications that this is generally a (cost)effective approach to prevention although it has not yet been studied for this specific group (Beekman ATF, Cuijpers P, van Marwijk HWJ, et al, Prevention of psychiatric disorders [In Dutch: Preventie van psychiatrische stoornissen.]. Ned Tijdschr Geneeskd 2006;150:419-23.).
We have started to pilot the intervention with two families (VUmc), and the first reactions were very positive. As demonstrated in the landmark studies of Mittelman et al., family meetings, designed to mobilize support of naturally existing family networks, appear to be among the most powerful psychosocial interventions to reduce depression in caregivers (Whitlatch 1991, Mittelman 1995, 1996, 2004).

8.5 Compensation for injury

We obtained dispensation from the statutory obligation to provide insurance, because participating in the study is without risks for informal caregivers and patients.

9 ADMINISTRATIVE ASPECTS AND PUBLICATION

9.1 Handling and storage of data and documents

The privacy of the participants will be guaranteed by coding the data so they can not be converted to the identity of the patients and caregivers. Therefore the 'indirect identifying' method will be used (Samenvatting Code Goed Gedrag van de FMWV). Only the primary investigators will have access to the source data. The data will be kept at most 5 years after publication (ibid.). The (video)tapes of the family meetings we use for training of the counselors, intervision and supervision, will be destroyed within a period of at most 5 years after the end of the study.

9.2 Amendments

All amendments will be notified to the METC-VUmc. Non-substantial amendments will not be notified to the METC and the competent authority, but will be recorded and filed by the sponsor.

9.3 Annual progress report

The investigator will submit a summary of the progress of the trial to the accredited METC once a year. Information will be provided on the date of inclusion of the first subject, numbers of subjects included and numbers of subjects that have completed the trial, serious adverse events/ serious adverse reactions, other problems, and amendments.

9.4 End of study report

The investigator will notify the METC-VUmc of the end of the study within a period of 8 weeks. The end of the study is defined as the moment the analysis and reports are completed (see planning and time schedule).
In case the study is ended prematurely, the investigator will notify the METC-VUmc, including the reasons for the premature termination. Within one year after the end of the study, the investigator/sponsor will submit a final study report with the results of the study, including any publications/abstracts of the study, to the METC-VUmc.

9.5 Public disclosure and publication policy

Each year the investigators will contact ZonMw about the progress that has been made. At the end of the study the final report will be sent to ZonMw within a period of 4 months.

REFERENCES

- AARP, National Alliance for Caregiving: Family Caregiving in the US: Findings from a national survey. Bethesda, MD, 1997.
- Acton GJ, Kang J: Interventions to reduce the burden of caregiving for an adult with dementia: a meta-analysis. Res Nurs Health 2001, 24: 349-360.
- Akkerman RL, Ostwald SK. Reducing anxiety in Alzheimer's disease family caregivers: the effectiveness of a nine-week cognitive-behavioral intervention. Am J Alzheimers Dis Other Demen. 2004;19:117-23.
- APA Diagnostic Statistical Manual IV of the American Psychiatric Association Washington 1994
- Ashworth M, Robinson S, Godfrey E, Sheperd M, Evans C, Seeley J: Measuring mental health outcomes in primary care: the psychometric properties of a new patients-generated outcome measure, 'PSYCHLOPS' ('psychological outcome profiles'). *Primary Care Mental Health* 2005, 3: 261-270.
- Beck A et al. An inventory for measuring clinical anxiety: psychometric properties. J Consult Clin Psychol. 1988;56(6):893-7
- Beekman A et al. Anxiety and depression in later life: Co-occurrence and communality of risk factors. Am J Psychiatry. 2000;157(1):89-95.
- Brandt J, Spencer M, Folstein M. The telephone interview for cognitive status. Neuropsychiatry Neuropsychol Behav Neurol 1988;1:111-17.
- Brodaty H, Green A, Koschera A. Meta-analysis of psychosocial interventions for caregivers of people with dementia. J Am Geriatr Soc. 2003;51:657-64.
- Cohen D. Caregivers for persons with Alzheimer's disease. Curr Psychiatry Rep. 2000;2:32-9.
- Cohen J, Statistical power analysis for the behavioural sciences. Academic press, New York/London 1977.
- Cuijpers P Depressive disorders in caregivers of dementia patients: a systematic review. Aging Ment Health. 2005;9:325-30.
- Cuijpers P. Examining the effects of prevention programs on the incidence of new cases of mental disorders: the lack of statistical power. Am J Psychiatry. 2003;160(8):1385-91.
- Derogatis LR, Lipman RS, Covi L. SCL-90: an outpatient psychiatric rating scale-preliminary report. Psychopharmacol.Bull. 1973;9(1):13-28.
- Drummond MF, Mohide EA, Tew M, et al: Economic evaluation of a support program for caregivers of demented elderly. Int J Technol Assess Health Care 1991;7: 209-219.
- Efron B, Tibshirani RJ. An introduction to the bootstrap. New York London: Chapman & Hall, 1993.
- Gaugler JE, Zarit SH, Pearlin LI. Family involvement following institutionalization: modeling nursing home visits over time. Int J Aging Hum Dev. 2003;57:91-117.
- Gitlin LN et al. Caregiver appraisals of functional dependence in individuals with dementia and associated caregiver upset: psychometric properties of a new scale and response patterns by caregiver and care recipient characteristics. J Aging Health. 2005;17:148-71.
- Given CW, Given B, Stommel M, Collins C, King S, Franklin S: The caregiver reaction assessment (CRA) for caregivers to persons with chronic physical and mental impairments. *Res Nurs Health* 1992, 15: 271-283.
- GR, Gezondheidsraad (Health Council of the Netherlands), dementie, Den Haag 2002

- Hofman A, Rocca WA, et al.: The prevalence of dementia in Europe: a collaborative study of 1980-1990 findings. Eurodem Prevalence Research Group. Int J Epidemiol 1991, 20: 736-748.
- Hoyl MT, Alessi CA, Harker JO, Josephson KR, Pietruszka FM, Koelfgen M et al.: Development and testing of a five-item version of the Geriatric Depression Scale. J Am Geriatr Soc 1999, 47: 873-878.
- Jang,Y., Clay, O.J., Roth, D.L., Haley, W.E. &. Mittelman, M.S. (2004). Neuroticism and longitudinal change in caregiver depression: Impact of a spouse-caregiver intervention program. The Gerontologist, 44, 311-317.
- Jong-Gierveld, J. de and F. Kamphuis: 1985, The development of a Rasch-Type Loneliness Scale, Applied Psychological Measurement 9, 289–299.
- Lyketsos CG et al. The life chart interview: a standardisde method to describe the course of psychopathology. Int J Meth Psychiatr Res 4: 143-155
- Mark A, Melanie S, Jeremy C, Veronica M, Kevin W, Henk P: A client-generated psychometric instrument: The development of PSYCHLOPS. Counselling and Psychotherapy *Research* 2004, 4: 27-31.
- Marks IM et al. Brief standard self-rating for phobic patients. Behav Res Ther. 1979;17(3):263-7
- Martikainen J, Valtonen H, Pirttila T: Potential cost-effectiveness of a family-based program in mild Alzheimer's disease patients. Eur J Health Econ 2004, 5: 136-142.
- Mitrani VB, Feaster DJ, et al. Adapting the structural family systems rating to assess the patterns of interaction in families of dementia caregivers. Gerontologist. 2005;45:445-55.
- Mittelman MS, Epstein C, Pierzchala A: Counseling the Alzheimer's Caregiver: A Resource for Health Care Professionals. geen idee: American Medical Association; 2003.
- Mittelman MS, Ferris SH, Shulman E, et al. A family intervention to delay nursing home placement of patients with Alzheimer disease. A randomized controlled trial. JAMA 1996, 276: 1725-1731.
- Mittelman, M.S., Roth, D.L., Coon D.W. and Haley, W.E. (2004). Sustained benefit of supportive intervention for depressive symptoms in Alzheimer's caregivers. American Journal of Psychiatry, 161(5), 850-856.
- Mittelman, M.S., Roth, D.L., Haley, W.E. & Zarit, S.H. (2004). Effects of a caregiver intervention on negative caregiver appraisals of behavior problems in patients with Alzheimer's disease: Results of a randomized trial. Journals of Gerontology, Psychological Sciences, 59B(1), P27-P34.
- Oostenbrink JB, Koopmanschap MA, Rutten FF. Handbook for cost studies, methods and guidelines for economic evaluation in health care. Health Care Insurance Council 2000.
- Oostenbrink JB, Koopmanschap MA, Rutten FF. Standardisation of costs: the Dutch Manual for Costing in economic evaluations. Pharmacoeconomics 2002;20:443-54.
- Ory MG, Hoffman R, Yee JL, ...Schulz R. Prevalence and impact of caregiving: a detailed comparison between dementia and nondementia caregivers. Gerontologist 1999;39:177-85.
- Ostwald SK, Hepburn KW, Caron W, Burns T, Mantell R. Reducing caregiver burden: a randomized psychoeducational intervention for caregivers of persons with dementia. Gerontologist. 1999;39:299-309.
- Ostwald SK. et al. Reducing caregiver burden: a randomized psychoeducational intervention for caregivers of persons with dementia. Gerontologist. 1999;39:299-309.
- Pearlin LI, Schooler C: The structure of coping. J Health Soc Behav 1978, 19: 2-21.

- Polder JJ ; Achterberg PW Cost of illness in the Netherlands RIVM Rapport, Bilthoven 2004 www.rivm.nl / www.costofillness.nl
- Pot, A.M., van Dyck, R., en D.J.H. Deeg (1995). Ervaren druk door informele zorg; constructie van een schaal. In: *Tijdschrift voor Gerontologie en Geriatrie* (1995) 26, p. 214-219.
- Pot AM, Deeg DJ, van Dyck R, Jonker C. Psychological distress of caregivers: the mediator effect of caregiving appraisal. Patient Educ Couns. 1998;34:43-51.
- Radloff L: The CES-D Scale: a Self -Report Depression Scale for Research in the General Population. Applied Psychological Measurement 1977, 1: 385-401.
- RIVM Rijksinstituut voor Volksgezondheid en Milieu. www.rivm.nl/Kostenvanziekten
- Robinson-Whelen S, Tada Y, MacCallum RC, McGuire L, Kiecolt-Glaser JK. Long-term caregiving: what happens when it ends? J Abnorm Psychol. 2001 Nov;110(4):573-84.
- Roth, D.R., Mittelman, M.S., Clay, O.J., Madan, A., Haley, W.E. (2005). Changes in social support as mediators of the impact of a psychosocial intervention for spouse caregivers of persons with Alzheimer's disease. Psychology and Aging, 20(4), 634-644.
- Rush AJ et al. The Inventory of Depressive Symptomatology (IDS): psychometric properties. Psychol Med. 1996;26(3):477-86.
- Schulz R, Beach SR: Caregiving as a risk factor for mortality: the Caregiver Health Effects Study. JAMA 1999, 282: 2215-2219.
- Schulz R, O'Brien AT, Bookwala J, Fleissner K: Psychiatric and physical morbidity effects of dementia caregiving: prevalence, correlates, and causes. Gerontologist 1995, 35: 771-791.
- SCP, Sociaal Culturaal Planbureau. Mantelzorg, over hulp van en aan mantelzorgers 2003
- Sheehan DV, Lecrubier Y, Sheehan KH, Amorim P, Janavs J, Weiller E, Hergueta T, Baker R, Dunbar GC. The Mini-International Neuropsychiatric Interview (M.I.N.I.): the development and validation of a structured diagnostic psychiatric interview for DSM-IV and ICD-10. J Clin Psychiatry. 1998;59 Suppl 20:22-33;quiz 34-57.
- Sorensen S, Pinquart M, Duberstein P: How effective are interventions with caregivers? An updated meta-analysis. Gerontologist 2002, 42: 356-372.
- Spinhoven P, Ormel J, Sloekers PP, Kempen GI, Speckens AE, Van Hemert AM: A validation study of the Hospital Anxiety and Depression Scale (HADS) in different groups of Dutch subjects. Psychol Med 1997, 27: 363-370.
- STG, Stuurgroep Toekomst Scenario's. Zorgen voor geestelijke gezondheidszorg in de toekomst; toekomstscenario's geestelijke volksgezondheid en geestelijke gezondheidszorg 1990-2010.
- Verhey F et al. Memory clinics in the Netherlands. Stockholm Meeting of the International Psychogeriatric Association 2005
- Verkaik R, Handboek voorkom somberheid met plezierige activiteiten. NIVEL Utrecht 2005
- Vernooij-Dassen MJ, Felling AJ, Brummelkamp E, Dauzenberg MG, van den Bos GA, Grol R. Assessment of caregiver's competence in dealing with the burden of caregiving for a dementia patient: a Short Sense of Competence Questionnaire (SSCQ) suitable for clinical practice J Am Geriatr Soc. 1999 Feb;47(2):256-7.
- Ware J, Kolinski M, Keller S: *How to score the SF-12 Physical and mental health summaries: a user's manual.* Boston: The Health Institute, New England Medical Centre; 1995.

- Whitlatch CJ, Zarit SH, von-Eye A. Efficacy of interventions with caregivers: A reanalysis. Gerontologist 1991;31:9-14.
- Wimo A, Ljunggren G, Winblad B: Costs of dementia and dementia care: a review. Int J Geriatr Psychiatry 1997, 12: 841-856.
- Wimo A, Wallin JO, Lundgren K, Ronnback E, Asplund K, Mattsson B et al.: Impact of day care on dementia patients--costs, well-being and relatives' views. Fam Pract 1990, 7: 279-287.
- Yesavage JA. Geriatric Depression Scale. Psychopharmacol.Bull. 1988;24(4):709-711.
- Zigmond AS, Snaith RP: The hospital anxiety and depression scale. Acta Psychiatr Scand 1983, 67: 361-370.
